# Increased Accuracy to c-Fos-Positive Neuron Counting

**DOI:** 10.1155/2021/3060983

**Published:** 2021-11-08

**Authors:** Wellington José da Silva, José Rodrigo Santos Silva, Jullyana de Souza Siqueira Quintans, Waldecy de Lucca Junior

**Affiliations:** ^1^Laboratory of Molecular Neuroscience of Sergipe (LaNMSE), Department of Morphology, Federal University of Sergipe, Av. Marechal Rondon, S/N, Rosa Elze, 49000-100 São Cristóvão, Sergipe, Brazil; ^2^Department of Statistic, Federal University of Sergipe, Av. Marechal Rondon, S/N, Rosa Elze, 49000-100 São Cristóvão, Sergipe, Brazil; ^3^Laboratory Neurosciences and Pharmacological Tests, Department of Physiology, Federal University of Sergipe, Av. Marechal Rondon, S/N, Rosa Elze, 49000-100 São Cristóvão, Sergipe, Brazil

## Abstract

There is not a described method to count the core label of c-Fos-positive neurons, avoiding false-positive and false-negative results. The aim of this manuscript is to provide guidelines for a secure and accurate method to calculate a threshold to select which core of c-Fos-positive neurons marked by immunofluorescence has to be scored. A background percentage was calculated by dividing the intensity value (0 to 255) of the core of c-Fos-positive neurons by its surrounding background from the 8-bit images obtained in a previous study. Using the background percentage from 20% up to 98%, raising 2% once for each score, as threshold to choose which core has to be counted, a script was written for the R program to count the number of the c-Fos-positive neurons and the comparison between control and experimental groups. The differences of the average number of the core counted c-Fos-positive neurons between control and experimental groups, at all thresholds studied, showed a rising value related to an increase of the background percentage threshold as well as a decrease of its *p* value related to an increase of the threshold of background percentage. For the smallest thresholds (high intensity of label), the differences between groups are suppressed (false negative). However, for the biggest thresholds (nonspecific label), these differences are always the same (false positive). Therefore, to avoid the false-negative and the false-positive values, it was chosen as the threshold of 62% the inflection point of the linear regression, which is equally different from the biggest and smallest values of the differences between groups.

## 1. Introduction

Fos protein is composed of 380 amino acids that form a heterodimer with Jun to bind itself to the DNA and promote a variety of actions in the cell. It can be produced 15 minutes after stimulation because it is a product of the immediate early gene c-Fos [1]. Its expression is an indirect marker for neuronal activity because it is frequently expressed after the neurons spike an action potential [2, 3]. Likewise, a conditional fear can inhibit the c-Fos mRNA expression in the animals stimulated to produce Fos protein. Such suppression indicates the repression of neural activity [4].

Immunofluorescence to Fos protein is a widespread procedure used by neuroscientists to study both excitatory [2, 3] and inhibitory [4] activations in the neural circuits [2, 5–7]. It has been used in qualitative studies to show the activation of the neurons in the central nervous system after a specific stimulus [8, 9]. Besides, it has also been used in quantitative studies, in which the number of c-Fos-positive neurons is counted to evaluate the recruitment of the neurons in the neural circuit nuclei. These quantitative studies have been used to evaluate the action of new medicines in the central nervous system, mainly to include a test to analgesic approach [10–14].

The methods used to core count c-Fos-positive neurons are not well described in the literature. The vast majority of the works do not provide details on the method used to core count c-Fos-positive neurons [6, 15].

To count the core label of c-Fos-positive neurons in the immunofluorescence images, it is necessary to overcome two issues: the variability of the core label intensity between different images (outside variability) and the gradient of the core label intensity inside of each image (inside variability). To control the influence of both the outside and the inside variabilities during the counting process of the core labels of c-Fos-positive neurons, there is not a described method capable of avoiding the false-positive and false-negative results. Aiming to fill that gap in the literature, this manuscript provides a secure and accurate method to count the core label of c-Fos-positive neurons marked by immunofluorescence.

## 2. Materials and Methods

### 2.1. Experimental Protocol

From a previous study [16], images of the experimental and control groups were selected to be used on the core count protocol proposed by this study. The core label of c-Fos-positive neurons was counted using several thresholds, from 20% up to 98%, raising 2% once, to compare control and experimental groups. After that, it was applied the inflection point of the linear regression, which is equally different from the biggest and smallest values of the differences between groups.

### 2.2. Image Acquisition

The 8-bit images were acquired with a CM10 camera of the IX81 Olympus microscopy, using 20x objective and fluorescence filter (U-FBWA) to Alexa Fluor 488. The frequency of the opening of the diaphragm was kept at 2 seconds.

### 2.3. Image Analysis

On each acquired image from the ventrolateral region of the PAG [16], the core of c-Fos-positive neurons was clicked, as well as the background around it, using a Cell Counter (Written by Kurt De Vos) plug-in for Image J software (NIH, USA). Subsequently, the intensity value (0 to 255) of the core of c-Fos-positive neurons was obtained, as well as the background around it, by clicking on the “Measure” button of the Cell Counter plug-in. These clicks on the core label and the background around it on the acquired image occurred, even when the core was overly weakly labeled. After that, the intensity value of the background around the core label was divided by the intensity value of the core label. As the intensity value of the core label was always higher than the intensity value of the background around it, the quotient obtained by that division was expressed by a percentage value which represents the background percentage of the core label. Therefore, the background percentage represents the quotient between the intensity of the background label (around the core label) and the intensity of the core label. Then, the R program was used to run a script, to write for the authors, to calculate the background percentage, and to count the marked c-Fos-positive neurons on the all thresholds (from 20% up to 98%, raising 2% once, of the background percentage) proposed in this study.

### 2.4. Statistical Analysis

All the calculations were made using the R statistic program [17]. The data obtained were expressed as the mean ± standard error of the mean, and the differences were evaluated through the Wilcoxon test [18]. Those with a *p* value less than 0.05 were considered as significant.

## 3. Results

**Figure 1:**
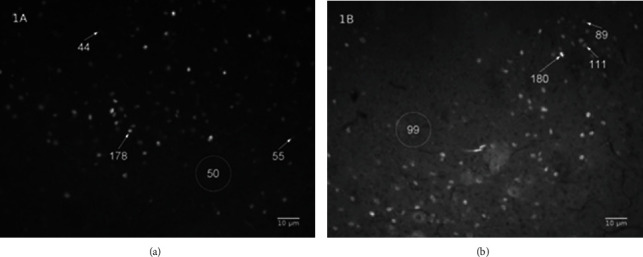
The variability of the intensity label of the core label of c-Fos-positive neurons. Photomicrography (200x) of the periaqueductal gray showing the core label of c-Fos-positive neurons of the two animals ((a, b)) submitted (both) to the treatment with MEKR (400 mg/kg; p.o. in the previous study [16]). The numbers below the arrows represent the intensity value of the core label of c-Fos-positive neurons. The values inside the circle represent the average of the intensity value of the background in the selected area limited by the circle.

**Figure 2:**
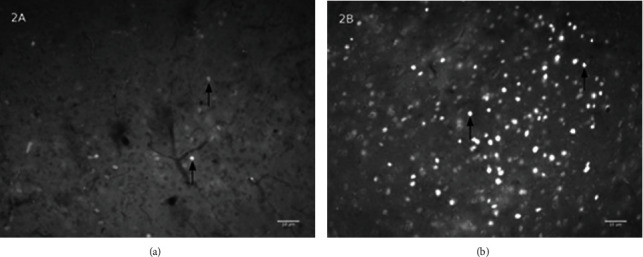
The effect of MEKR treatment in c-Fos expression. Photomicrography (200x) of the periaqueductal gray showing core label (arrows) of c-Fos-positive neurons after treatment with MEKR (b) or vehicle (a).

The background of image 1(b) has an intensity value (around 99) bigger than several core labels of image 1(a) (55 and 44). These differences of the intensity value between these images (1(a) and 1(b)) characterize the outside variability. In Figure 1, the intensity value of the core label of c-Fos-positive neurons inside picture 1(a) ranges from 44 to 178, and its background is around 50. This gradient of the core label intensity characterizes the inside variability, at some parts of the image 1(a); the smallest intensity label of the core can be smaller than its background label. Because of the variability (outside and inside), this work proposes to use the background percentage, obtained by division of the intensity value of the background image by core label, as a threshold to choose which core has to be scored. It was used several background percentages as a threshold, from 20% up to 98%, raising 2% once, to count the core label of the c-Fos-positive neurons (Figure 2).

Figure 2 shows the c-Fos-positive neurons (arrows) on the PAG in the mouse brain after either vehicle (a) or MEKR (b) treatment. The number of c-Fos-positive neurons activated on the PAG is raised by the MEKR (*p* < 0.05) treatment, as demonstrated in previous research by our group [16]. Using the script below, the number of c-Fos-positive neurons was counted.

**Figure 3:**
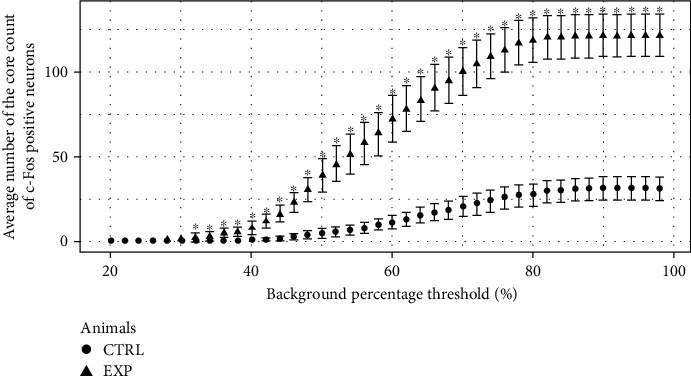
Counts of the core label of c-Fos-positive neurons in the periaqueductal gray. The average number of the core label scored according to background percentage on periaqueductal gray on control (Ctrl) and experimental (Exp) groups.

In Figure 3, the average number of the core counts of the c-Fos-positive neurons in the PAG for all calculated background percentage thresholds (from 20% up to 98%, raising 2%) was plotted. Between control and experimental groups shows, on this plot, a rise in the difference of the average number of the core counts for c-Fos-positive neurons related to the increase of the background threshold, except for both extremes of the curves. For the smallest thresholds (high intensity of label), the differences between groups are suppressed (false negative). However, for the biggest thresholds (nonspecific label), these differences are always the same (false positive).

**Figure 4:**
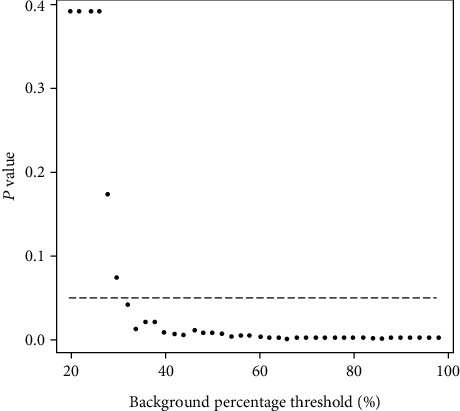
Variation of the *p* value on Wilcoxon test. Variation of the *p* value according to background percentage on Wilcoxon test applied to compare control groups against experimental groups.

After evaluating the core count average number, the value of *p* on the Wilcoxon test for the differences between control and experimental groups at all thresholds was studied (Figure 4). It was observed a decrease in the *p* values related to the increase in the threshold of the background percentage.

**Figure 5:**
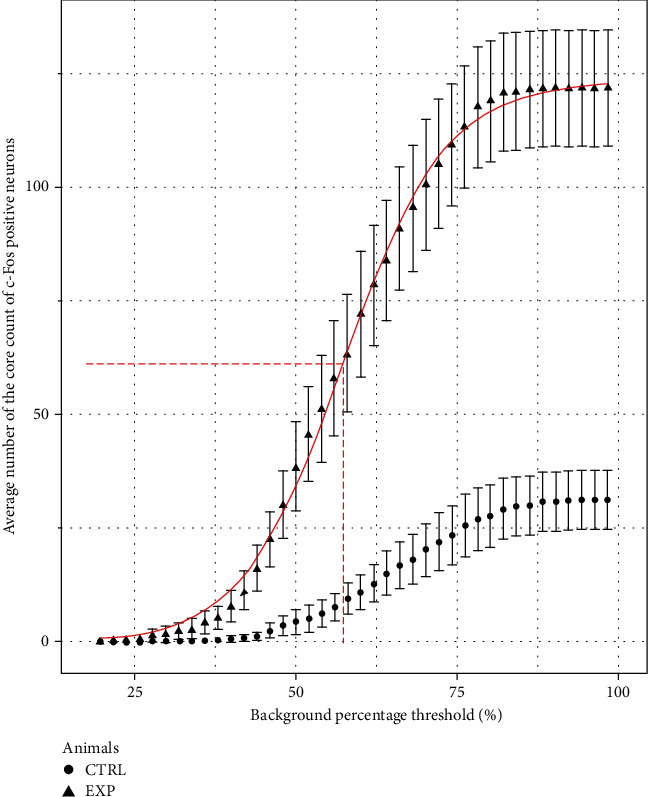
Counts of the core label of c-Fos-positive neurons in the periaqueductal gray. Average number of the core label counted to control (Ctrl) groups and experimental (Exp) groups, using a threshold of 62% (inflection point) to background around the core label. The difference is significant on the Wilcoxon test.

Therefore, to avoid the false-negative and the false-positive values, it was chosen as the threshold of 62% (Figure 5). This value represents the inflection point of the linear regression, which is equally different from the biggest and smallest values of the differences between groups.

## 4. Discussion

The proposed method in this manuscript uses mathematical language to calculate a safe threshold to the core count of c-Fos-positive neurons marked by immunofluorescence.

The results demonstrated that, for the weak core labels, the background percentage showed high intensity values (around 80%). On the other hand, for the strong core labels, the background percentage showed low intensity values (around 20%). Hence, to select which core label had to be counted, the background percentage was used as a threshold. Thereby, many counts of the core label were made with a threshold from 20% to 98%, raising 2% once. For each threshold, the amount of the core label of c-Fos-positive neurons for the control was measured, as well as for the experimental groups. Moreover, the *p* value was calculated using a Wilcoxon test and analyzed whether there was a significant difference between the groups studied.

In Figure 3, that difference between control and experimental groups was observed because the increase on the average number of the core counted c-Fos-positive neurons was more intense in experimental than in the control group. This was an expected result because the control group does not have many c-Fos-positive neurons, whereas the experimental group has a lot of c-Fos-positive neurons. This higher core counts in the experimental group were due to the pharmacological effect produced by MEKR [16].

For thresholds ranging from 20% to 30%, the average number of the core count was not significantly different, whether it was compared to the control against the experimental groups (Figure 3), and the *p* value was higher than 0.05 (Figure 4). For these threshold levels, only a few cores are counted in the experimental group because there are not many neurons with extraordinarily high intensity of the core label. These results indicate that counting only neurons with high intensity of the core label can provoke a type II error, i.e., a “false-negative” result.

For thresholds from 78% to 98% (Figure 3), the average number of the core count showed a significant difference between control and experimental groups with an extremely small *p* value (*p* < 0.001). However, the comparison inside the same group, either the control group or the experimental group, did not show differences in the average number of the core count. The probable reason for it is the weak stain of the core label for this threshold range. For a weak stain, it is difficult to differentiate the core label of the background around it. In Figure 1(a), the intensity of the background percentage around the core label was indicated by a circle for different thresholds. The core label cannot be clearly differentiated from the background around it for thresholds from 78% to 98%. Therefore, using this threshold range makes it hard to differentiate specific from nonspecific binding from the artifact labels. These difficulties can provoke a type I error, i.e., a “false-positive” result.

“False-negative” results are equally damaging to science as “false-positive” results, since both can generate a cascade of errors producing consequences that are often incalculable [19].

To choose which threshold should be the most indicated for the core count, a linear correlation was made, and the results showed its inflection point as threshold 62% in Figure 5.

In the literature, there are several methods to core count c-Fos-positive neurons. The most frequently used consists of counting cells by clicking on them, using the common sense to establish a threshold to separate the background from the specific label [6]. This method cannot be safe if the differences between experimental and control groups are tiny.

In our method to core count c-Fos-positive neurons, we also click on the core label of the neurons to count c-Fos-positive neurons. Thus, all core labels are clicked, even if the core label is not so intense. However, unlike the traditional methods, our method resides at the decision of which core has to be counted and which does not. In our method, a mathematical threshold is established to decide which core has to be counted and which does not. That way, all nucleus cells are clicked, but not all of them are counted. Just the cores with a background intensity of 62% or lower than the intensity of the core label is counted.

Other works have used a threshold limit with a fixed value [8, 20] to choose which cores have to be counted and which does not. That is a good strategy, but using the same threshold for all immunofluorescence pictures cannot be so efficient because the value of the background usually has a considerable variation, even if camera's diaphragm is kept at the same frequency during the acquisition process. Because the method described in this paper uses a percentage background of the label as value, not a fixed value, all variation in the intensity of the core label can be normalized, avoiding “false-positive and false-negative” results.

The proposed method allows to evaluate more accurately the core count of c-Fos-positive neurons marked by immunofluorescence, allowing a reduction of the number of animals.

This manuscript offers an additional tool to improve the production of neuroscience studies, for which animals and humans will benefit.

## 5. Conclusions

Figure 5 shows that for background percentage values greater than 62%, there is no increase in the difference between the experimental and control groups. Therefore, 62% is the maximum threshold that allows counting the c-Fos-positive neurons safely. From that, considering the varying average number of the core label of c-Fos-positive neurons and the behavior of the *p* value of Wilcoxon test on the threshold of the background studied, it should be advisable to use a threshold of 62% of the background percentage (Figure 5) to select which cores has to be counted.

## Figures and Tables

**Code 1:**
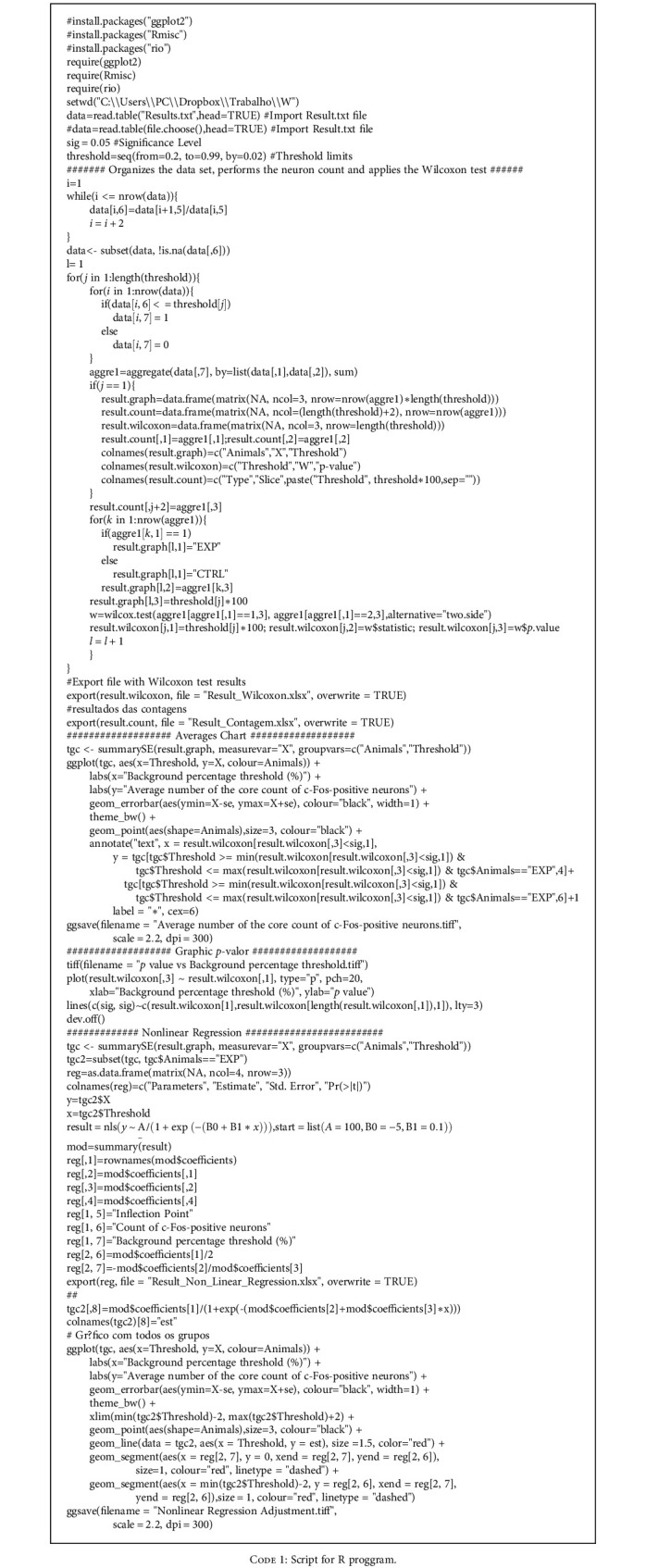
Script for R proggram.

## Data Availability

The script, source images, and data used to support the findings of this study have been deposited in http://www.lb.ufs.br/threshold/.
